# Investigating Individual and Familial Stress on Alzheimer’s Disease among a Global Cohort of Non‐Hispanic Blacks and Hispanic/Latinos

**DOI:** 10.1002/alz.093470

**Published:** 2025-01-09

**Authors:** Rhoda Moise, Dingtian Cai, Farid Rajabli, Kara L. Hamilton‐Nelson, Larry D. Adams, Michael L. Cuccaro, Brian W. Kunkle, Jeffery M. Vance, Christiane Reitz, Giuseppe Tosto, William S. Bush, Jonathan L. Haines, Goldie S. Byrd, Rufus O. Akinyemi, Adesola Ogunniyi, Susan Halloran Blanton, Margaret A Pericak‐Vance, Azizi A Seixas, Africa Dementia Consortium

**Affiliations:** ^1^ Department of Psychiatry and Behavioral Sciences, University of Miami Miller School of Medicine, Miami, FL USA; ^2^ John P. Hussman Institute for Human Genomics, University of Miami Miller School of Medicine, Miami, FL USA; ^3^ Dr. John T. Macdonald Foundation Department of Human Genetics, University of Miami Miller School of Medicine, Miami, FL USA; ^4^ University of Miami, Miller School of Medicine, John P. Hussman Institute for Human Genomics, Miami, FL USA; ^5^ Dr. John T. Macdonald Foundation Department of Human Genetics, Miller School of Medicine, University of Miami, Miami, FL USA; ^6^ Gertrude H. Sergievsky Center, Taub Institute for Research on the Aging Brain, Departments of Neurology, Psychiatry, and Epidemiology, College of Physicians and Surgeons, Columbia University, New York, NY USA; ^7^ Department of Population and Quantitative Health Sciences, Institute for Computational Biology, Case Western Reserve University, Cleveland, OH USA; ^8^ Maya Angelou Center for Health Equity, Wake Forest University School of Medicine, Winston‐Salem, NC USA; ^9^ Neuroscience and Aging Research Unit, Institute of Advanced Medical Research and Training, College of Medicine, University of Ibadan, Ibadan Nigeria; ^10^ Africa Dementia Consortium, Ibadan, Ibadan Nigeria; ^11^ College of Medicine, University of Ibadan, Ibadan, Oyo State Nigeria; ^12^ Department of Informatics and Health Science Data, University of Miami Miller School of Medicine, Miami, FL USA

## Abstract

**Background:**

Stress has emerged as a risk factor in the development and progression of Alzheimer’s Disease (AD). However, there is limited research on the impact cumulative individual and familial stress has on AD. This study aims to investigate the relationship between stress and AD within a global cohort of underrepresented populations including Black Americans (BAs), Hispanic/Latinos (H/Ls), and Africans (As).

**Method:**

The total sample size was N = 328. Binomial regression in R examined the stress‐AD association, controlling for age, sex, race/ethnicity, and site. Stress was defined as “a situation in which a person feels tense, restless, nervous, or anxious, or is unable to sleep at night because his/her mind is troubled all the time.” Individuals were asked 1) Do you feel this kind of stress these days? and 2) While growing up did you and your family experience stress? Participants rated stress on a 0‐4 Likert scale. Binary recoding distinguished low (not at all) and high stress (all else). Ethnicity was coded as H/Ls and Non‐Hispanic Black Utilizing the binomial family, the analysis robustly explored the relationship between stress and AD diagnosis.

**Results:**

A quarter of the sample (mean age = 73.53, SD = 8.63) had AD, while most had no cognitive impairment (mean age = 71.13, SD = 6.88). 73% were females (N = 240). Low‐stress individuals are 1.812 times more likely to be non‐cognitively impaired (p = 0.03767). Familial stress is not significant (p = 0.83255). Although there were significant results for ethnic group and age, their interpretation captures the distribution of cases and controls within those groups. Our experimentation was not designed to test them, but the inclusion of these variable in the model serve to adjust for the main variable of interest, stress.

**Conclusion:**

This study reveals the stress‐AD link in a diverse population, emphasizing the necessity of targeted interventions considering individual stress in cognitive health research, urging further exploration of familial stress and ethnicity nuances. Understanding these relationships is crucial for tailored support systems and preventive strategies. Future research should delve deeper into stress as a modifiable AD risk factor, exploring underlying mechanisms for a comprehensive understanding. Additionally, future studies should design to further explore age and ethnicity in AD prediction.

